# The decline of FGM in Egypt since 1987: a cohort analysis of the Egypt Demographic and Health Surveys

**DOI:** 10.1186/s12905-020-00954-2

**Published:** 2020-05-11

**Authors:** Ronan Van Rossem, Dominique Meekers

**Affiliations:** 1 Department of Sociology, Universiteit Gent, Korte Meer 3-5, 9000 Ghent, Belgium; 2 School of Public Health & Tropical Medicine, Department of Global Community Health and Behavioral Sciences, Tulane University, 1440 Canal Street, Suite 2200, New Orleans, LA 70112 USA

**Keywords:** Female genital mutilation, Egypt, Reproductive health, women’s status, Empowerment

## Abstract

**Background:**

Female genital mutilation (FGM) has been a longstanding tradition in Egypt and until recently the practice was quasi-universal. Nevertheless, there are indications that the practice has been losing support and that fewer girls are getting cut. This study analyzes the prevalence of FGM in different birth cohorts, to test whether the prevalence declined over time. The study also examines whether such a decline is occurring in all segments of society or whether it is limited mostly to certain more modernized segments of society.

**Methods:**

This study pooled data from the 2005, 2008 and 2014 waves of the Egypt Demographic and Health Surveys (EDHS). The women participating in the EDHS provided data on 62,507 girls born to them between 1987 and 2014, including whether they were cut and at what age. Kaplan-Meier and Weibull proportional hazard survival analyses were used to examine trends in the prevalence and hazards of FGM across birth cohorts. Controls for region, religion and socioeconomic status of the parents were included in the Weibull regression.

**Results:**

The results show a steady decline in FGM across the birth cohorts studied. The base hazard for the 2010 birth cohort is only 30% that of the 1987 one. Further analyses show that the decline in FGM occurred in all segments of Egyptian society in a fairly similar manner although differences by region, religion and socioeconomic status persisted.

**Conclusions:**

This study confirms that FGM is declining in Egypt. The proportion of girls getting cut has declined rapidly over the past few decades. This decline is not limited to the more modernized segments of society, but has spread to the more traditional segments as well. The latter increases prospects for the eventual eradication of the practice.

Egypt has a long history of female genital mutilation (FGM) and the practice is firmly embedded in Egyptian culture and tradition. Historically FGM had the support of almost the entire population and almost all girls were cut. The United Nations Children’s Fund (UNICEF) [1] reported that in 2016 Egypt was ranked 6th out of 29 countries in terms of the prevalence of FGM. Only Somalia, Guinea, Djibouti, Sierra Leone and Mali have higher prevalence rates. In Egypt, types I and II [2] are the most common forms of FGM, while types III and IV are quite rare [3, 4]. A recent study [5] found that 74% of the women had Type I FGM and 26% Type II. However, as most prevalence data are for adult women they reflect practices of decades ago. There are, however, indications that support for FGM is diminishing and that the practice is declining. Once such decline has set in, it may progress rapidly. For instance, in 2013 UNICEF estimated the prevalence of FGM among women aged 14 through 49 in Egypt at 91% [6], but by 2016 the estimate had lowered to 87% [7, 8]. According to the 2014 EDHS 92% of ever married women between the ages of 15 and 49 were circumcised [9]. However, among 20–24-year-old ever married women it was only 87%, while among 35 to 49-year-olds it was 95%. El-Gibaly, Ibrahim, Mensch and Clark [10] also demonstrated that the prevalence of FGM among girls aged 10–19 is about 10 percentage points lower than among their mothers. This paper uses data from three nationally representative *Egypt Demographic and Health Surveys* (EDHS) to examine the extent to which the prevalence of FGM has changed across the birth cohorts from 1987 to 2014. Using data on whether the daughters of the women participating in the EDHS were cut, and at which age they were cut, will provide information on more recent evolutions regarding FGM in Egypt.

## Background

FGM is deeply embedded in a society’s culture and tradition and intimately related to women’s social status and honor [11–17]. The behavior of women, and their sexual behavior in particular, reflects not only on their own honor but also that of their family. In such a context it is important to families to guard the virginity and reputation of daughters until marriage. For instance, in Egypt the loss of virginity prior to marriage is often considered a disgrace that may even lead to honor killings [18]. To this end, families and communities tend to strictly control the social and sexual behavior of women by restrictive norms on their social participation, particularly their interactions with the other sex. These norms can be manifested through strict dress codes, and, in some societies, FGM [13, 19, 20].

However, although these customs reinforce the subordinate status of women they also provide status to women. Being cut symbolizes that a woman is of good standing, which tends to increase her marriageability, social status and prestige [13, 21–28]. Non-cut women, to the contrary, run the risk of being socially excluded and to be perceived as immoral. Parents who desire the best for their daughters are therefore under substantial pressure to have their daughters cut. Not having one’s daughter cut may endanger her future by lowering both her own social status and that of her family [17, 19, 23] . The groups best situated to go in against tradition are typically those that have alternative sources of social status, those that have least to loose, and those that live in less traditional social environments. It therefore will be primarily families from the higher and more modern social strata that will not have their daughters cut, as well as groups at the lower end of or outside the traditional status hierarchy, such as the very poor and religious minorities.

International organizations promote increasing female empowerment, improving women’s position in society, and reducing gender inequality as a strategy to eradicate FGM [11]. Typically, such organizations focus on improving both the education levels of women and their labor force participation in modern economic sectors and thus reducing their dependency and enhancing their agency [29, 30], i.e., to provide women with alternative sources of social status and make them less dependent on traditional sources. Wealthier, better educated and urban women are indeed more likely to oppose FGM, and are also less likely to intend to have their daughters cut [10, 24, 31–38].

In Egypt gender inequality is high. The United National Development Programme’s (UNDP) *Gender Inequality Index* declined slightly between 1995 and 2015, from 0.665 to 0.565, but it remains high and progress has been slower than in most other countries [39]. El-Safety [40] reports that there has been a conservative backlash that led to a de facto curtailment of women’s rights. Moreover, women’s progress has not been equal across all domains. For instance, while Egyptian women made considerable progress in terms of education, little or no progress was made regarding labor force participation and employment. Literacy levels among women age 15 and older increased from 22.4% in 1976 to 67.2% in 2013, while gross secondary school enrollment of women increased from 21.4% in 1971 to 85.7% in 2014. The percentage of women aged 15 and older who are employed, to the contrary, remained low: 26.7% in 1990 and only 23.0% in 2016 [41].

There is ample evidence that social support for FGM in Egypt has declined substantially over the past few decades [33, 34, 42–49]. Until recently, a large majority of the population supported FGM [15, 50]. In 2003, only 23.3% of ever-married women favored its discontinuation, while 60.8% believed that FGM was required by their religion [48]. Opposition to FGM increased between 1995 and 2014 [see: 34, 49]. In 1995 only 13% of ever married women believed the practice of FGM should be stopped, but by 2014 this had increased to 31% [34]. Despite gradually increasing opposition to FGM, the overwhelming majority of women have undergone FGM and still support it [49]. Although attitudinal change is not sufficient for behavior change, it can be a necessary precursor to behavior change [51, 52]. People are more likely to abandon traditional behaviors when such behaviors are delegitimized while alternative ones gain acceptance.

Delegitimation of FGM occurs first and most pronounced in both the more modern segments of society (i.e., among those who are better educated, urban, or employed in modern sectors), as well as among the least traditional segments of Egyptian society (i.e., non-Muslims) [34]. Opposition to FGM subsequently spreads to the other segments of society [33, 34]. Some studies suggest that opposition to FGM in Egypt spread rapidly during the first decade of this century, but somewhat less rapidly in recent years [9, 43–46, 48].

The ongoing legislative actions against FGM may be another factor contributing to its delegitimation. For several decades the Egyptian authorities have tried to curb and regulate FGM. In 2007 and 2008 laws were passed that banned the practice [53, 54]. However, it remains unclear how rigidly these laws are enforced, especially given the political upheavals since 2011. Although the 2007 law prohibited general practitioners from performing FGM, Rasheed, Abd-Ellah and Yousef [55] found that the incidence of FGM in Upper Egypt remained very high, and that most cuttings were still performed by general practitioners. As in most other countries, anti-FGM legislation reportedly only had limited direct impact because “[…] of sporadic enforcement of the laws, lack of comprehensive legislation, lack of robust institutions to enforce the law and programs aimed at addressing the underlying social norms that perpetuate the practice” [56]. Nevertheless, legislative changes may gradually undermine the legitimacy of the practice and may have an effect in the long term.

This study examines the prevalence of FGM and its evolution over a range of birth cohorts (1987–2014). We expect the risk of being cut to decline in more recent birth cohorts [Hypothesis 1]. However, even within birth cohorts there are variations in the risk of FGM. Modernized and marginalized groups function as innovators and will typically be the first to decide not to have their daughters cut. Girls born to better educated or higher socioeconomic status (SES) parents or from more marginalized (e.g., non-Muslim) families will be less likely to be cut than those born in more traditional families [Hypothesis 2]. As anti-FGM attitudes diffuse throughout society, the risk of being cut is expected to decline in other segments of Egyptian society, thereby reducing the difference between the innovative groups and the rest of society, except for some lagging segments of society [Hypothesis 3].

## Methods

### Datasets

This paper uses data from the 2005, 2008 and 2014 EDHS [57–59]. The EDHS are conducted under the auspices of the Egyptian Ministry of Health and Population with technical support from the Demographic and Health Surveys (DHS) program which is sponsored by United States Agency for International Development (USAID). Although the sampling procedures differed slightly across surveys, they all used a multistage sampling procedure: 1) the primary sampling units (PSU) (shiakhas/villages) are sampled with a probability proportional to size; 2) the PSU were divided in sectors of about 1000 households or 5000 inhabitants which were sampled systematically; 3) each sector was split up in segments of about equal size which were sampled either systematically or with a probability proportional to the size of the PSU; 4) within each selected segment households were sampled using systematic random sampling and 5) in these households all ever married women between the ages of 15 and 49 were eligible to participate in the survey. The overall response rate was extremely high with 98.5% for the 2005 EDHS, 98.8% for the 2008 EDHS and 97.8% for the 2014 EDHS. Survey staff received extensive training to assure the quality of the data collection. For more detailed information on the survey design and data collection, we refer to the study documentation [9, 46, 48].

The target population of the EDHS surveys consists of ever-married women aged 15 through 49. The pooled sample size of the three surveys used is *N* = 57,763 (*N*_2005_ = 19,474, *N*_2008_ = 16,527, and *N*_2014_ = 21,762). However, the unit of analysis of our study consists of the women’s surviving daughters aged 0–19, about whom data about FGM was collected. The pooled surveys contain data on *N*_*d*_ = 62,507 daughters (*N*_*d*,2005_ = 20,190, *N*_*d*,2008_ = 18,287, and *N*_*d*,2014_ = 23,310, all unweighted) from *N*_*m*_ = 37,409 mothers (*N*_*m*,2005_ = 12,478, *N*_*m*,2008_ = 10,734, and *N*_*m*,2014_ = 14,197). This implies that the mothers in the sample had an average of 1.67 daughters; this varies little across survey waves (2005: 1.68, 2008: 1.70, 2014: 1.64). Most women (54.5%) only have a single daughter, 29.8% have two daughters, 11.2% 3, and only 4.5% four or more daughters. The procedures and questionnaires of all DHS surveys have been reviewed and approved by the *ICF International* institutional review board (IRB) and comply with the *U.S. Department of Health and Human Services* regulations for the protection of human subjects (45 CFR 46); they have also been reviewed by an Egyptian IRB to assure compliance with Egyptian rules and laws [34]. The de-identified EDHS data are publicly available from https://dhsprogram.com.

### Variables

The dependent variable for this study is duration of exposure to the risk of FGM. For girls who experienced FGM, this variable equals the age (in years) at which they experienced FGM. For non-cut girls, it equals their age at time of the survey. A dichotomous status variable indicates whether the girl was cut. The year of birth of the girls was used to define the cohorts, and ranges from 1987 to 2014. For some analyses cohorts were combined in five-year intervals: 1987–1990, 1991–1995, 1996–2000, 2001–2005, 2006–2010, and 2011–2014. All confounding variables are measured at the level of the mother. These include the region where the mother lives, her religion, education level and occupation, as well as the education level and occupation of her spouse. In nearly all cases, the latter will be the girl’s biological father, but if the girl’s mother remarried, it may be her social father. For simplicity, we will refer to this person as the girl’s father.

### Statistical analysis

To track the evolution of FGM by age for each birth cohort Kaplan-Meier estimators are used with the larger five-year birth cohorts as stratifying variable. For the most recent birth cohorts the available data is limited to the younger ages, as the entire survival function cannot be estimated. Multilevel Weibull proportional hazard regression techniques are used to analyze the effects of the confounding variables on the risk of being cut. The regression coefficients represent the changes in the underlying log-hazard, i.e., they are partial log-hazard ratios. As before, for the younger birth cohorts only part of the survival function can be estimated. All analyses were conducted using the sampling weights provided by the EDHS surveys.

## Results

### Univariate

Table 1 shows descriptive statistics for the daughters in the three EDHS waves, separately as well as pooled. Given that the EDHS only collects data on children 0–19 years, each of the three waves included in the analysis covers a different range of birth cohorts. Pooled, the most frequent birth cohorts are those born between 1996 and 2000 and between 2001 and 2005 (25 and 27%, respectively). Earlier and later birth cohorts are less well represented in the final sample.

**Table 1:** Descriptive statistics, by EDHS wave

	EDHS wave			
	2005	2008	2014	Total
Weighted N	20,851	18,164	23,491	62,506
Cohort				***
1987–1990	17.6%	10.0%	0.0%	8.8%
1991–1995	25.2%	21.9%	5.1%	16.7%
1996–2000	29.3%	26.0%	19.7%	24.7%
2001–2005	27.8%	27.7%	24.3%	26.5%
2006–2010	0.0%	14.5%	28.8%	15.0%
2011–2014	0.0%	0.0%	22.2%	8.3%
Region				***
Urban governorates	14.6%	15.8%	11.5%	13.8%
Urban LE	9.7%	10.8%	10.1%	10.1%
Rural LE	31.0%	32.5%	36.1%	33.4%
Urban UE	12.8%	10.9%	11.4%	11.7%
Rural UE	30.6%	28.4%	30.1%	29.8%
Frontier governorates	1.3%	1.5%	1.0%	1.2%
Mother’s education				***
No education	39.7%	36.7%	26.7%	33.9%
Incomplete primary	11.7%	9.7%	6.8%	9.3%
Complete primary	4.2%	4.1%	4.6%	4.3%
Incomplete secondary	9.1%	10.6%	12.4%	10.8%
Complete secondary	26.7%	29.8%	37.4%	31.6%
Higher	8.6%	9.2%	12.1%	10.1%
Mother’s occupation				***
Not working	76.6%	82.8%	83.3%	80.9%
Professional, Technical, Managerial.	7.6%	7.4%	7.6%	7.5%
Clerical	2.5%	1.7%	1.5%	1.9%
Sales	1.8%	0.9%	2.0%	1.6%
Agriculture-self employed	4.0%	0.6%	1.1%	1.9%
Agriculture-employee	3.4%	2.5%	1.9%	2.6%
Services	2.1%	2.4%	1.5%	1.9%
Skilled manual	1.1%	0.7%	1.0%	0.9%
Unskilled manual	0.9%	1.1%	0.2%	0.7%
Don’t know	0.0%	0.0%	0.0%	0.0%
Mother’s religion				***
Muslim	95.0%	95.8%	96.5%	95.8%
Christian	5.0%	4.2%	3.4%	4.1%
Father’s education				***
No education	25.2%	24.2%	18.3%	22.3%
Primary	20.9%	18.6%	15.0%	18.0%
Secondary	39.5%	42.9%	51.8%	45.1%
Higher	14.5%	14.3%	14.9%	14.6%
Father’s occupation				***
Not working	3.4%	3.2%	2.3%	3.0%
Professional, Technical, Managerial.	22.9%	24.0%	22.0%	22.9%
Clerical	5.7%	4.5%	4.6%	4.9%
Sales	3.4%	2.2%	4.8%	3.6%
Agriculture-self employed	9.8%	7.7%	5.9%	7.7%
Agriculture-employee	8.2%	10.7%	8.7%	9.1%
Services	11.6%	14.2%	12.1%	12.5%
Skilled manual	24.8%	26.1%	30.9%	27.5%
Unskilled manual	9.3%	6.9%	8.3%	8.2%
Don’t know	0.9%	0.5%	0.4%	0.6%

Significance: *: *p* < 0.050, **: *p* < 0.010, ***: *p* < 0.001

The girls’ mothers also have become better educated over time. In the 2005 EDHS about 40% of the girls have an uneducated mother. In the 2014 EDHS this proportion has declined to 27%, while the proportion of girls with mothers with complete secondary or higher education has increased from 35 to 50%. Although better education for women is often seen as evidence of the improvement of women’s social position, in this case this improvement is clearly only partial as the labor market participation of the mothers remains poor and may even be worsening. In 2005 77% of the girls have non-working mothers, compared to 83% in 2014. More than 95% of the girls also have Muslim mothers. Pooled data from the three EDHS waves show that only 22% of girls have an uneducated father, while 60% have a father who has secondary or higher education. Only 3% of girls have a father who is not working and the two most prevalent occupational categories are skilled manual laborers (28%) and professional, technical or managerial occupations (23%).

### Evolution of FGM over the birth cohorts

**Fig. 1:**
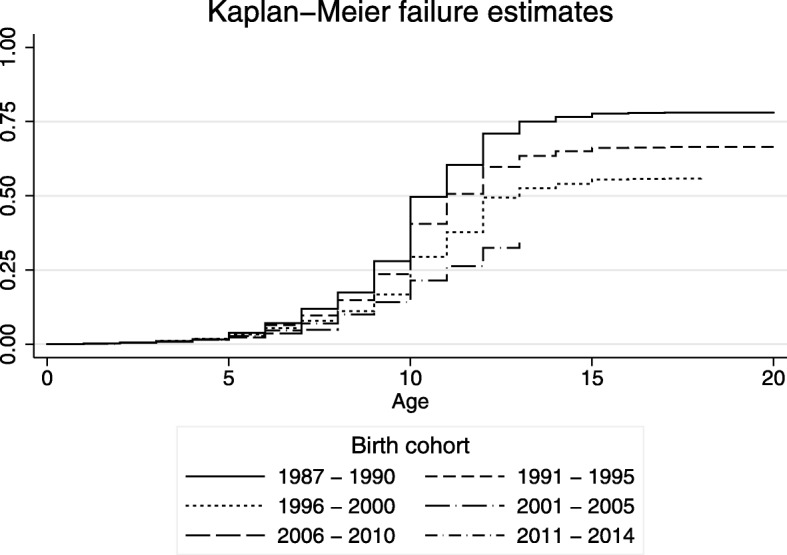
Estimated proportions of women who experienced FGM, by age and birth cohort

**Fig. 2:**
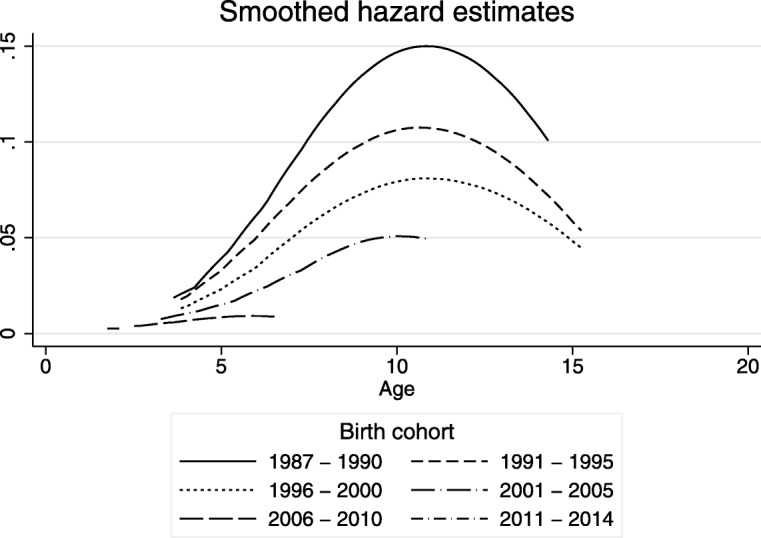
Estimated hazards of FGM, by age and birth cohort

Figure 1 shows the Kaplan-Meier estimates of the proportion of girls who underwent FGM by age for the different birth cohorts. The estimated prevalence of FGM at any given age is always lower in each subsequent cohort. For example, the estimated percentage of girls who were cut by age 19 decreased from 78% for the 1987–1990 birth cohort to 67% for the 1991–1995 cohort. The estimated proportion of girls cut by age 15 decreased from 77% in the 1987–1990 birth cohort to 66% for 1991–1995 and to 54% for 1996–2000. Similarly, the percentage of girls who were cut by age 10 decreased from 50% for the 1987–1990 birth cohort to 41% for 1991–1995, and further to 21% for 2001–2005. As only few girls get cut after the age of 13 these trends show a swift decline in the percentage of girls that undergo FGM in Egypt over the past few decades. Figure 2 shows the estimated hazard rates for being cut at each age for the different birth cohorts. The findings show that the risk of being cut declines at all ages across birth cohorts. The overall shape of the hazard functions remains similar for each of the different cohorts, with a maximum hazard at about age 10. However, in each subsequent birth cohort the hazard at each age is lower than in the previous birth cohort. Figures S1 through S6 show that FGM is declining among all segments of Egyptian society.

### Regression models

**Fig. 3:**
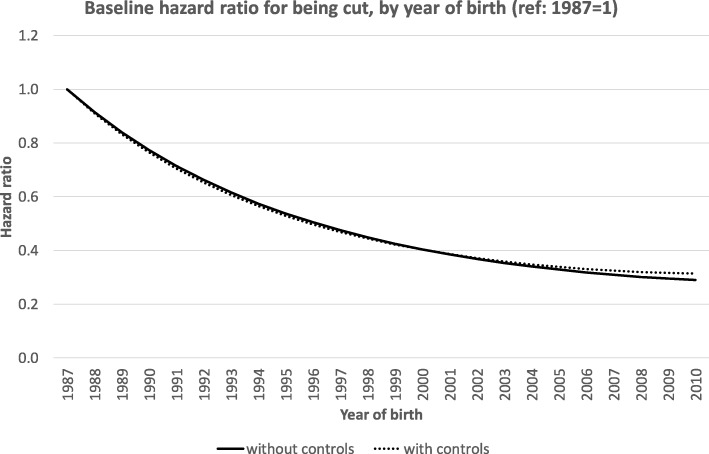
Estimated changes in the hazard for FGM, Weibull distribution, by birth cohort (1987 = 1), and with and without controlling for mother and mother’s partner characteristics

Figure 3 shows the baseline hazard ratios for FGM by the year of birth relative to girls in 1987, i.e., the evolution of the hazard for FGM across all groups and ages. The results confirm the declining risk of being cut among the more recent birth cohorts compared to the 1987 birth cohort. There is a rapid decline in FGM among the 1990s birth cohorts; among the cohorts born after 2000 the risk of FGM continues to decline but at a somewhat slower pace. By definition, the birth cohort reference hazard ratio equaled 1 for 1987; the estimated hazard ratio declined to 0.40 for the 2000 birth cohort and to 0.29 for the 2010 cohort. Controlling for background characteristics of the mothers does not affect the baseline hazard ratios by birth cohort.

Table 2 shows the Weibull proportional hazard results for the effect of confounding variables on age at FGM. The unadjusted effects shown in Model 1 confirm that girls from more recent birth cohorts have a lower hazard of experiencing FGM. Model 2 shows the main effects of the background characteristics on the hazard of being cut, independent from the overall trend in FGM. On average, girls living in the urban governorates run the lowest risk of experiencing FGM while those living in Upper Egypt tend to have the highest risk. The average estimated hazard for girls living in Urban Upper Egypt is 12.12 times as high as for those living in the Urban governorates, and in Rural Upper Egypt the hazard ratio (HR) even equaled 18.52, and this while controlling for the other confounding variables (both *p* < 0.001). The hazard ratio (HR) equals the antilog of the regression coefficients in Table 2, or $HR\;(X)={e}^{b_x}$, where *HR*(*X*) equals the HR associated with factor *X* and *b*_*X*_ the Weibull regression coefficient for factor *X*. In Rural Lower Egypt the estimated HR is 3.25 (*p* < 0.001). The educational level of the mother is another important factor affecting girls’ risk of being cut. Girls born to better educated mothers are significantly less likely to be cut than those born to mothers without any formal education. The HR for girls born to uneducated mothers compared to girls born to mothers with a complete secondary education is only 0.36, while for those born to mothers with higher education it is only 0.10 (both *p* < 0.001). Noteworthy is that for girls born to mothers with an incomplete secondary education the hazard of being cut is slightly but significantly higher than for those born to mothers with no formal education (HR = 1.14, *p* < 0.050). The labor market status of the mother also affects the risk of being cut. On average girls born to working mothers have a lower hazard of being cut than those born to non-working mothers. The only exception consists of girls born to mothers working in professional, technical or managerial jobs, whose hazard of being cut does not significantly differ from those born to non-working mothers. We also expected FGM to decline in the least traditional segments of society. The results show that girls born to Christian mothers are at a significantly lower risk of being cut than those born to Muslim mothers (HR = 0.13, *p* < 0.001).

**Table 2:** Weibull proportional hazard survival analysis results for age at FGM

*b* (se)	(1)	(2)
Year of birth	−0.090*** (0.009)	−0.095*** (0.009)
(Year of birth)^2^	0.002** (0.001)	0.002*** (0.001)
Region of residence (ref: Urban governorates)		
Urban LE		0.165* (0.067)
Rural LE		1.178*** (0.055)
Urban UE		2.495*** (0.068)
Rural UE		2.919*** (0.063)
Frontier governorates		0.391*** (0.093)
Mother’s education (ref: No education)		
Incomplete primary		−0.069 (0.042)
Complete primary		−0.039 (0.067)
Incomplete secondary		0.131* (0.052)
Complete secondary		−1.021*** (0.051)
Higher		−2.337*** (0.113)
Mother’s religion (ref: Muslim)		
Christian		−2.056*** (0.107)
Mother’s occupation (ref: Not working)		
Prof., Tech., Manag.		0.077 (0.074)
Clerical		−0.385*** (0.105)
Sales		−0.516*** (0.100)
Agric-self employed		−0.309*** (0.087)
Agric-employee		−0.539*** (0.069)
Services		−0.461*** (0.098)
Skilled manual		−0.266* (0.118)
Unskilled manual		−0.565*** (0.144)
Don’t know		1.122 (1.103)
Father’s education (ref: No education)		
Primary		−0.182*** (0.039)
Secondary		0.057 (0.042)
Higher		−0.368*** (0.074)
Father’s occupation (ref: Not working)		
Prof., Tech., Manag.		−0.018 (0.077)
Clerical		0.044 (0.089)
Sales		−0.058 (0.108)
Agric-self employed		−0.290*** (0.079)
Agric-employee		−0.381*** (0.078)
Services		0.068 (0.076)
Skilled manual		−0.170* (0.071)
Unskilled manual		−0.191* (0.081)
Don’t know		−0.358 (0.210)
Constant	−17.138*** (0.289)	−18.423*** (0.309)
ln(*p*)	1.902 (0.015)	1.931 (0.015)
${\hat{\sigma}}_u^2$	9.249 (0.315)	7.984 (0.277)

Significance: *: *p* < 0.050, **: *p* < 0.010, ***: *p* < 0.001

The father’s background also affects whether a girl gets cut, independently from the mother characteristics, but these effects are considerably weaker than those of the mother’s characteristics. Girls whose father has either only primary education or has higher education have a lower hazard of being cut compared to when the father has no formal education, while if the father had secondary education the hazard of being cut does not differ significantly from those with no formal education. Regarding the occupation of the father, only girls whose father was working in agriculture had significant lower hazards of being cut, compared to those whose father was not working.

**Fig. 4:**
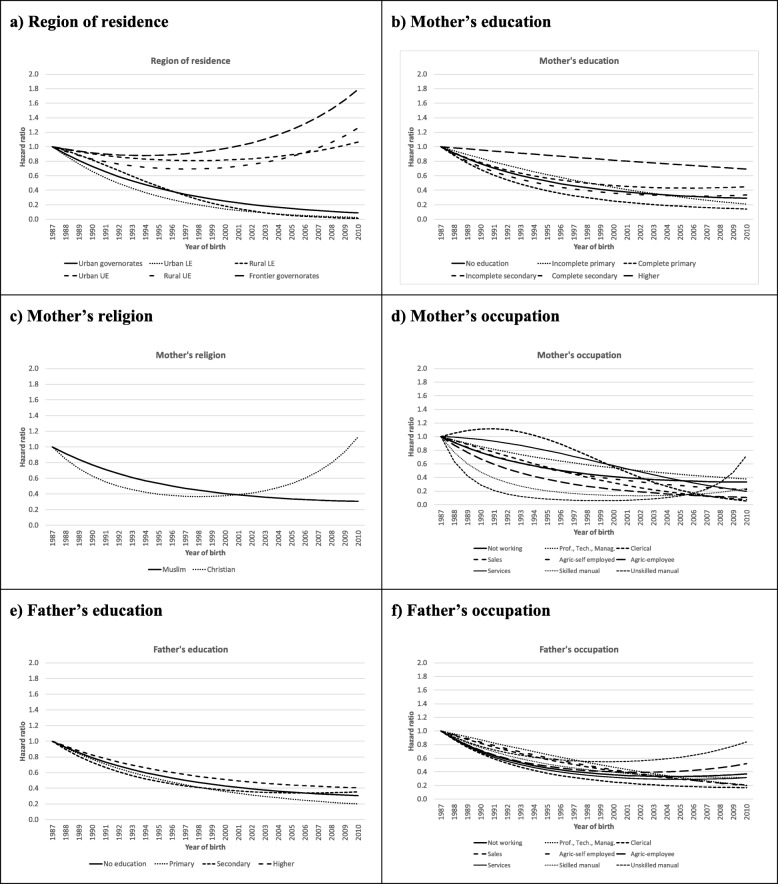
Estimated hazard ratios for selected factors, by year of birth

Subsequent analyses examined whether the decline of the hazard for FGM is equal for all segments of society or whether certain groups lead the decline while others lag. The results are shown in Fig. 4 and in models 3 through 8 in Table S1. The differences in the evolutions of the hazards by background characteristics were estimated by including the interaction terms between the background characteristics and the girls’ year of birth (linear and squared) in the Weibull proportional hazard regression, controlling for the main effects of the other confounding variables. The estimates for the youngest birth cohorts are less efficient as these are relatively small cohorts with a shorter duration of exposure to the risk of FGM than the older cohorts. Figure 4 shows the estimated relative hazard ratios for the various categories of the confounding variables by birth cohort compared to the 1987 birth cohort (HR = 1).

For three of the confounding variables, mother’s religion, mother’s and father’s education, no significant interactions with the girl’s year of birth were observed, implying that the trend in the risk of FGM did not differ across the various categories of these variables and that they all followed the trend previously observed in Fig. 3. However, differences in the hazards for FGM remain, as the main effects of these variables all remain significant.

For the other three confounding variables (region of residence, mother’s and father’s occupation), significant interactions with the girl’s year of birth were observed, even after controlling for the other confounding variables, implying that the different categories not only differ in the level of risk but also experience different trends in the evolution of this risk. Although the pairwise comparisons of the trends for the different regions did not show any significant differences, Fig. 4a suggests that Upper Egypt and the Urban Governorates experienced a much slower decline than the other regions, and even a stagnation of the risk of FGM. Differences in the trends are also observed by the occupation of the mother. The hazard of a girl being cut declines more slowly among women occupied in clerical jobs compared to non-working mothers. Among skilled and unskilled manual laborers the initial decline is much faster, to subsequently stabilize at a low level. Likewise, for girls whose father is employed in a professional, technical or managerial occupation the hazard of being cut was somewhat slower to decline than for those whose father was not working. Although these differences in the trends of the HR are significant, they all tend to be quite small. For all categories the overall trend remains the same; the risk for FGM is substantially declining over the birth cohorts for all categories of the confounding variables.

## Discussion

The literature on FGM in Egypt suggests that the prevalence of FGM is declining and that an increasing proportion of girls no longer get cut [10, 32, 37, 47, 49, 55, 60, 61]. However, data on the prevalence of FGM among adult women reflect practices that took place years, or even decades ago. Our study is innovative because it examines changes in the age pattern of FGM (i.e. the probability of experiencing FGM by a given age) across cohorts born between 1987 and 2014. Our analyses show that the risk for FGM has been steadily declining since at least the 1990s. For instance, the estimated base hazard for the 2010 birth cohort was only 30% of that of the 1987 birth cohort. This is a substantial decline over a period of less than 25 years. The percentage of girls who experienced FGM by age 15 decreased from 77% for the 1987–1990 cohort to 66% for the 1991–1995 cohort, and further to 54% in the 1996–2000 cohort. The analyses suggest that this proportion will decline even further in more recent cohorts. The decline of FGM is quite universal and occurs among all segments of Egyptian society. This evolution cannot be explained by improvements in the position of women as in this case the decline should be concentrated in the more modernized segments of society. The universal decline suggests that other factors are at play. These may include anti-FGM campaigns, international pressures and anti-FGM legislation [62].

## Conclusions

The results of this paper suggest that FGM in Egypt will keep declining in the near future. In just a few decades the number of girls who were subjected to FGM has already declined substantially, and once a tipping point has been reached a further decline may occur quite fast. However, success in eradicating FGM in Egypt is by no means a certainty. The final eradication of the practice will require a continued effort, not only from the public authorities enforcing anti-FGM legislation, but also from anti-FGM advocates in civil society. Further improvements in women’s social position may also contribute to the decline of FGM. Unfortunately, although more women are getting secondary or higher educated, their labor force participation is still lagging. In addition to the need to improve women’s social positions, the eradication of FGM also requires a cultural shift that severs the links between a woman’s honor, her family’s status and FGM. Egypt has made serious strides in this direction, but by no means has it already won the war.

## Supplementary information

**Additional file 1: Table S1.** Weibull proportional hazard survival analysis results for age at FGM, including interaction terms between risk factors and birth cohort. **Figure S1.** Kaplan-Meier estimates for FGM by age, birth cohort, and region of residence. **Figure S2.** Kaplan-Meier estimates for FGM by age, birth cohort, and mother’s education. **Figure S3.** Kaplan-Meier estimates for FGM by age, birth cohort, and mother’s religion. **Figure S4.** Kaplan-Meier estimates for FGM by age, birth cohort, and mother’s occupation. **Figure S5.** Kaplan-Meier estimates for FGM by age, birth cohort, and father’s education. **Figure S6.** Kaplan-Meier estimates for FGM by age, birth cohort, and father’s occupation.

## Data Availability

The EDHS dataset are available, upon registration, from the DHS Program at https://dhsprogram.com. The dataset generated and analyzed during the current study are available in the OSF repository at DOI 10.17605/OSF.IO/EZKHQ.

## Abbreviations

DHS: Demographic and health surveys
EDHS: Egypt Demographic and health surveys
FGM: Female genital mutilation
HR: Hazard ratio
IRB: Institutional review board
PSU: Primary sampling unit
SES: Socio-economic status
UNDP: United national development programme
UNICEF: United Nations children’s fund
USAID: United States agency for international development
