# Supplementary material for: The decline of FGM in Egypt since 1987: a cohort analysis of the Egypt Demographic and Health Surveys
Source: BMC Womens Health. 2020 May 11;20:100. doi: 10.1186/s12905-020-00954-2 (PMC7216476; doi:10.1186/s12905-020-00954-2)
Supplement: Supplementary file 1 — Additional file 1: Table S1. Weibull proportional hazard survival analysis results for age at FGM, including interaction terms between risk factors and birth cohort. Figure S1. Kaplan-Meier estimates for FGM by age, birth cohort, and region of residence. Figure S2. Kaplan-Meier estimates for FGM by age, birth cohort, and mother’s education. Figure S3. Kaplan-Meier estimates for FGM by age, birth cohort, and mother’s religion. Figure S4. Kaplan-Meier estimates for FGM by age, birth cohort, and mother’s occupation. Figure S5. Kaplan-Meier estimates for FGM by age, birth cohort, and father’s education. Figure S6. Kaplan-Meier estimates for FGM by age, birth cohort, and father’s occupation. [file 12905_2020_954_MOESM1_ESM.pdf]

Table S1 Weibull proportional hazard survival analysis results for age at FGM, including interaction terms between risk factors and birth cohort

b<br>(se)	(3)	(4)	(5)	(6)	(7)	(8)	(9)
Daughter’s year of birth	-0.106**<br>(0.037)	-0.095***<br>(0.014)	-0.093***<br>(0.010)	-0.094***<br>(0.011)	-0.083***<br>(0.017)	-0.127**<br>(0.037)	-0.127*<br>(0.053)
(Daughter’s year of birth)^2	0.000<br>(0.002)	0.002*<br>(0.001)	0.002**<br>(0.001)	0.002**<br>(0.001)	0.001<br>(0.001)	0.004<br>(0.002)	0.000<br>(0.003)
Region of residence (ref: Urban governorates)	***	***	***	***	***	***	***
Urban LE	0.472**<br>(0.171)	0.166*<br>(0.068)	0.166*<br>(0.067)	0.168*<br>(0.067)	0.169*<br>(0.067)	0.163*<br>(0.067)	0.563**<br>(0.174)
Rural LE	1.284***<br>(0.132)	1.171***<br>(0.055)	1.178***<br>(0.055)	1.181***<br>(0.055)	1.176***<br>(0.055)	1.179***<br>(0.055)	1.213***<br>(0.136)
Urban UE	1.803***<br>(0.161)	2.488***<br>(0.068)	2.495***<br>(0.068)	2.496***<br>(0.068)	2.493***<br>(0.068)	2.502***<br>(0.068)	1.798***<br>(0.160)
Rural UE	2.325***<br>(0.133)	2.908***<br>(0.063)	2.918***<br>(0.063)	2.921***<br>(0.063)	2.916***<br>(0.063)	2.922***<br>(0.063)	2.196***<br>(0.138)
Frontier governorates	-0.288<br>(0.196)	0.382***<br>(0.094)	0.391***<br>(0.093)	0.392***<br>(0.093)	0.389***<br>(0.094)	0.391***<br>(0.093)	-0.350<br>(0.198)
Mother’s education (ref: No education)	***	***	***	***	***	***	***
Incomplete primary	-0.061<br>(0.042)	-0.177<br>(0.104)	-0.070<br>(0.042)	-0.069<br>(0.042)	-0.073<br>(0.042)	-0.075<br>(0.042)	-0.318**<br>(0.108)
Complete primary	-0.017<br>(0.068)	0.252<br>(0.188)	-0.038<br>(0.067)	-0.037<br>(0.067)	-0.038<br>(0.067)	-0.050<br>(0.067)	0.051<br>(0.190)
Incomplete secondary	0.125*<br>(0.052)	0.013<br>(0.143)	0.131*<br>(0.052)	0.132*<br>(0.052)	0.133*<br>(0.052)	0.130*<br>(0.052)	-0.207<br>(0.153)
Complete secondary	-0.969***<br>(0.052)	-0.949***<br>(0.109)	-1.021***<br>(0.051)	-1.021***<br>(0.051)	-1.020***<br>(0.051)	-1.020***<br>(0.051)	-1.151***<br>(0.142)
Higher	-2.244***<br>(0.113)	-2.840***<br>(0.234)	-2.338***<br>(0.113)	-2.342***<br>(0.113)	-2.338***<br>(0.113)	-2.328***<br>(0.113)	-2.970***<br>(0.292)

Mother's religion (ref: Muslim)	***	***	***	***	***	***	***
Christian	-2.017***	-2.047***	-1.900***	-2.055***	-2.055***	-2.057***	-1.657***
	(0.106)	(0.107)	(0.214)	(0.107)	(0.107)	(0.107)	(0.215)
Mother's occupation (ref: Not working)	***	***	***	***	***	***	***
Prof., Tech., Manag.	0.041	0.090	0.077	-0.112	0.084	0.067	-0.072
	(0.075)	(0.074)	(0.074)	(0.149)	(0.074)	(0.074)	(0.176)
Clerical	-0.453***	-0.396***	-0.384***	-0.762**	-0.381***	-0.402***	-0.948***
	(0.106)	(0.107)	(0.105)	(0.246)	(0.105)	(0.105)	(0.257)
Sales	-0.472***	-0.511***	-0.517***	-0.435	-0.519***	-0.512***	-0.465
	(0.101)	(0.100)	(0.099)	(0.261)	(0.100)	(0.100)	(0.260)
Agric-self employed	-0.300**	-0.306**	-0.308***	-0.282	-0.310***	-0.316***	-0.180
	(0.087)	(0.088)	(0.088)	(0.170)	(0.088)	(0.088)	(0.179)
Agric-employee	-0.537***	-0.536***	-0.539***	-0.226	-0.538***	-0.543***	-0.246
	(0.068)	(0.069)	(0.069)	(0.214)	(0.069)	(0.069)	(0.216)
Services	-0.512***	-0.459***	-0.461***	-0.738**	-0.456***	-0.463***	-0.931***
	(0.100)	(0.099)	(0.099)	(0.229)	(0.099)	(0.099)	(0.229)
Skilled manual	-0.254*	-0.260*	-0.263*	0.495	-0.264*	-0.275*	0.380
	(0.119)	(0.119)	(0.118)	(0.274)	(0.118)	(0.118)	(0.274)
Unskilled manual	-0.535***	-0.568***	-0.564***	0.795**	-0.571***	-0.549***	0.928**
	(0.143)	(0.144)	(0.144)	(0.299)	(0.144)	(0.144)	(0.311)
Don't know	1.186	1.214	1.120	-5.837	1.116	1.130	-6.278
	(1.175)	(1.118)	(1.103)	(7.962)	(1.105)	(1.107)	(7.603)
Father's education (ref: No education)	***	***	***	***	***	***	*
Primary	-0.187***	-0.183***	-0.182***	-0.181***	-0.090	-0.186***	-0.115
	(0.039)	(0.039)	(0.039)	(0.039)	(0.091)	(0.039)	(0.097)
Secondary	0.041	0.061	0.057	0.060	0.161	0.047	0.127
	(0.042)	(0.042)	(0.042)	(0.042)	(0.094)	(0.042)	(0.117)
Higher	-0.421***	-0.357***	-0.367***	-0.362***	-0.484**	-0.373***	-0.236
	(0.075)	(0.074)	(0.074)	(0.074)	(0.144)	(0.074)	(0.189)

Father's occupation (ref: Not working)	***	***	***	***	***	***	***
Prof., Tech., Manag.	-0.025	-0.013	-0.017	-0.025	-0.016	-0.263	-0.259
	(0.078)	(0.078)	(0.077)	(0.077)	(0.077)	(0.157)	(0.168)
Clerical	0.041	0.044	0.045	0.035	0.043	0.251	0.242
	(0.090)	(0.090)	(0.090)	(0.090)	(0.090)	(0.203)	(0.217)
Sales	-0.045	-0.051	-0.056	-0.067	-0.060	-0.188	-0.274
	(0.108)	(0.108)	(0.108)	(0.108)	(0.108)	(0.274)	(0.260)
Agric-self employed	-0.274**	-0.286***	-0.289***	-0.302***	-0.290***	-0.478**	-0.460**
	(0.080)	(0.079)	(0.079)	(0.079)	(0.079)	(0.170)	(0.175)
Agric-employee	-0.389***	-0.373***	-0.379***	-0.390***	-0.382***	-0.444**	-0.476**
	(0.078)	(0.078)	(0.078)	(0.078)	(0.078)	(0.169)	(0.173)
Services	0.062	0.072	0.069	0.057	0.067	0.139	0.092
	(0.076)	(0.076)	(0.076)	(0.076)	(0.076)	(0.165)	(0.165)
Skilled manual	-0.174*	-0.166*	-0.170*	-0.181*	-0.172*	-0.252	-0.390*
	(0.072)	(0.072)	(0.071)	(0.071)	(0.071)	(0.154)	(0.155)
Unskilled manual	-0.162*	-0.183*	-0.190*	-0.199*	-0.190*	-0.476**	-0.397*
	(0.082)	(0.082)	(0.081)	(0.081)	(0.082)	(0.182)	(0.182)
Don't know	-0.304	-0.361	-0.356	-0.361	-0.355	-1.800**	-1.554**
	(0.210)	(0.211)	(0.210)	(0.210)	(0.211)	(0.553)	(0.558)
Interaction: region of residence (ref: Urban governorates) & ...							
Daughter's year of birth							
Urban LE	-0.029						-0.061
	(0.058)						(0.057)
Rural LE	0.058						0.056
	(0.042)						(0.042)
Urban UE	0.067						0.054
	(0.045)						(0.044)
Rural UE	0.034						0.038
	(0.039)						(0.039)
Frontier governorates	0.069						0.062

	(0.056)		(0.056)
(Daughter's year of birth)^2	***		***
Urban LE	-0.001		0.001
	(0.004)		(0.004)
Rural LE	-0.006*		-0.006*
	(0.003)		(0.003)
Urban UE	0.002		0.003
	(0.003)		(0.003)
Rural UE	0.004		0.004
	(0.003)		(0.002)
Frontier governorates	0.003		0.004
	(0.004)		(0.003)
Interaction: Mother's education (ref: No education) & ...			
Daughter's year of birth			
Incomplete primary		0.039	0.056
		(0.032)	(0.032)
Complete primary		-0.038	-0.004
		(0.052)	(0.050)
Incomplete secondary		0.005	0.034
		(0.032)	(0.035)
Complete secondary		-0.023	-0.017
		(0.025)	(0.033)
Higher		0.080	0.057
		(0.056)	(0.072)
(Daughter's year of birth)^2			
Incomplete primary		-0.002	-0.002
		(0.002)	(0.002)
Complete primary		0.000	0.000
		(0.003)	(0.003)
Incomplete secondary		0.001	0.000
		(0.002)	(0.002)

Complete secondary	0.001			0.003
	(0.001)			(0.002)
Higher	-0.002			0.002
	(0.003)			(0.004)
Interaction: Mother's religion (ref: Muslim) & ...				
Daughter's year of birth				
Christian		-0.087		-0.101
		(0.056)		(0.056)
(Daughter's year of birth)^2				
Christian		0.006		0.005
		(0.003)		(0.003)
Interaction: Mother's occupation (ref: Not working)				
Daughter's year of birth			***	***
Prof., Tech., Manag.			0.041	0.034
			(0.039)	(0.046)
Clerical			0.154*	0.183*
			(0.077)	(0.073)
Sales			0.039	0.024
			(0.067)	(0.067)
Agric-self employed			0.007	-0.061
			(0.053)	(0.050)
Agric-employee			-0.043	-0.060
			(0.073)	(0.073)
Services			0.089	0.125*
			(0.061)	(0.061)
Skilled manual			-0.178*	-0.168
			(0.086)	(0.085)
Unskilled manual			-0.378***	-0.441***
			(0.092)	(0.106)
Don't know			0.314	0.316

	(0.920)		(0.876)
(Daughter's year of birth)^2	*		**
Prof., Tech., Manag.	-0.002		-0.001
	(0.002)		(0.003)
Clerical	-0.010		-0.010*
	(0.005)		(0.005)
Sales	-0.005		-0.002
	(0.004)		(0.004)
Agric-self employed	-0.001		0.004
	(0.004)		(0.003)
Agric-employee	0.000		0.002
	(0.005)		(0.005)
Services	-0.005		-0.006
	(0.003)		(0.003)
Skilled manual	0.007		0.008
	(0.005)		(0.005)
Unskilled manual	0.018**		0.023**
	(0.005)		(0.007)
Don't know	0.018		0.022
	(0.027)		(0.026)
Interaction: Father's education (ref: No education) & ...			
Daughter's year of birth			
Primary		-0.008	-0.011
		(0.026)	(0.028)
Secondary		-0.032	-0.032
		(0.024)	(0.029)
Higher		0.015	-0.062
		(0.034)	(0.046)
(Daughter's year of birth)^2			
Primary		0.000	0.000
		(0.002)	(0.002)

Secondary	0.002		0.002
	(0.001)		(0.002)
Higher	0.000		0.004
	(0.002)		(0.003)
Interaction: Father's occupation (ref: Not working) & ...			
Daughter's year of birth		**	**
Prof., Tech., Manag.		0.083*	0.111*
		(0.042)	(0.045)
Clerical		-0.017	-0.005
		(0.055)	(0.058)
Sales		0.060	0.071
		(0.069)	(0.067)
Agric-self employed		0.069	0.059
		(0.047)	(0.049)
Agric-employee		0.001	0.014
		(0.046)	(0.047)
Services		-0.010	0.003
		(0.046)	(0.046)
Skilled manual		0.026	0.056
		(0.042)	(0.042)
Unskilled manual		0.030	0.034
		(0.049)	(0.050)
Don't know		0.453***	0.447**
		(0.130)	(0.133)
(Daughter's year of birth)^2		**	**
Prof., Tech., Manag.		-0.005	-0.007**
		(0.003)	(0.003)
Clerical		-0.001	-0.002
		(0.003)	(0.003)
Sales		-0.004	-0.004
		(0.004)	(0.004)

Agric-self employed						-0.004	-0.003
						(0.003)	(0.003)
Agric-employee						0.001	0.000
						(0.003)	(0.003)
Services						0.000	-0.001
						(0.003)	(0.003)
Skilled manual						-0.001	-0.003
						(0.002)	(0.003)
Unskilled manual						0.000	-0.001
						(0.003)	(0.003)
Don't know						-0.026**	-0.028**
						(0.008)	(0.008)
Constant	-18.149***	-18.395***	-18.422***	-18.454***	-18.465***	-18.342***	-17.970***
	(0.324)	(0.309)	(0.309)	(0.309)	(0.312)	(0.326)	(0.342)
/ln_p	1.929	1.930	1.931	1.932	1.931	1.932	1.931
	(0.015)	(0.015)	(0.015)	(0.015)	(0.015)	(0.015)	(0.015)
IDNUM							
var(_cons)	7.791	7.941	7.971	8.003	7.973	8.008	7.771
	(0.270)	(0.276)	(0.277)	(0.278)	(0.277)	(0.278)	(0.269)
N	54,052	54,052	54,052	54,052	54,052	54,052	54,052
AIC	96,527.93	96,895.68	96,892.87	96,883.62	96,897.84	96,896.35	96,510.29

Significance: *: $p < 0.050$, **: $p < 0.010$, ***: $p < 0.001$

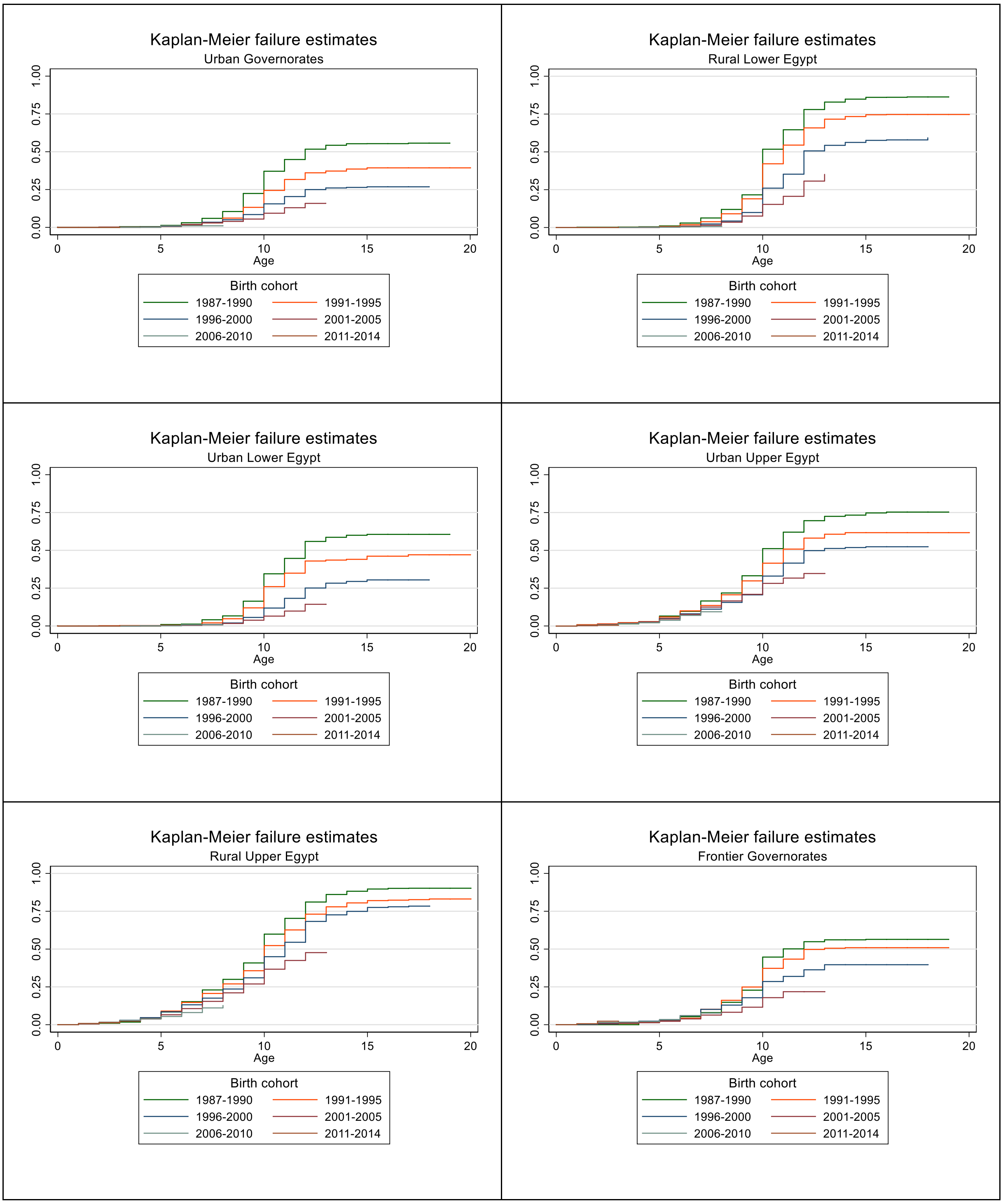

Fig. S1 Kaplan-Meier estimates for FGM by age, birth cohort, and region of residence

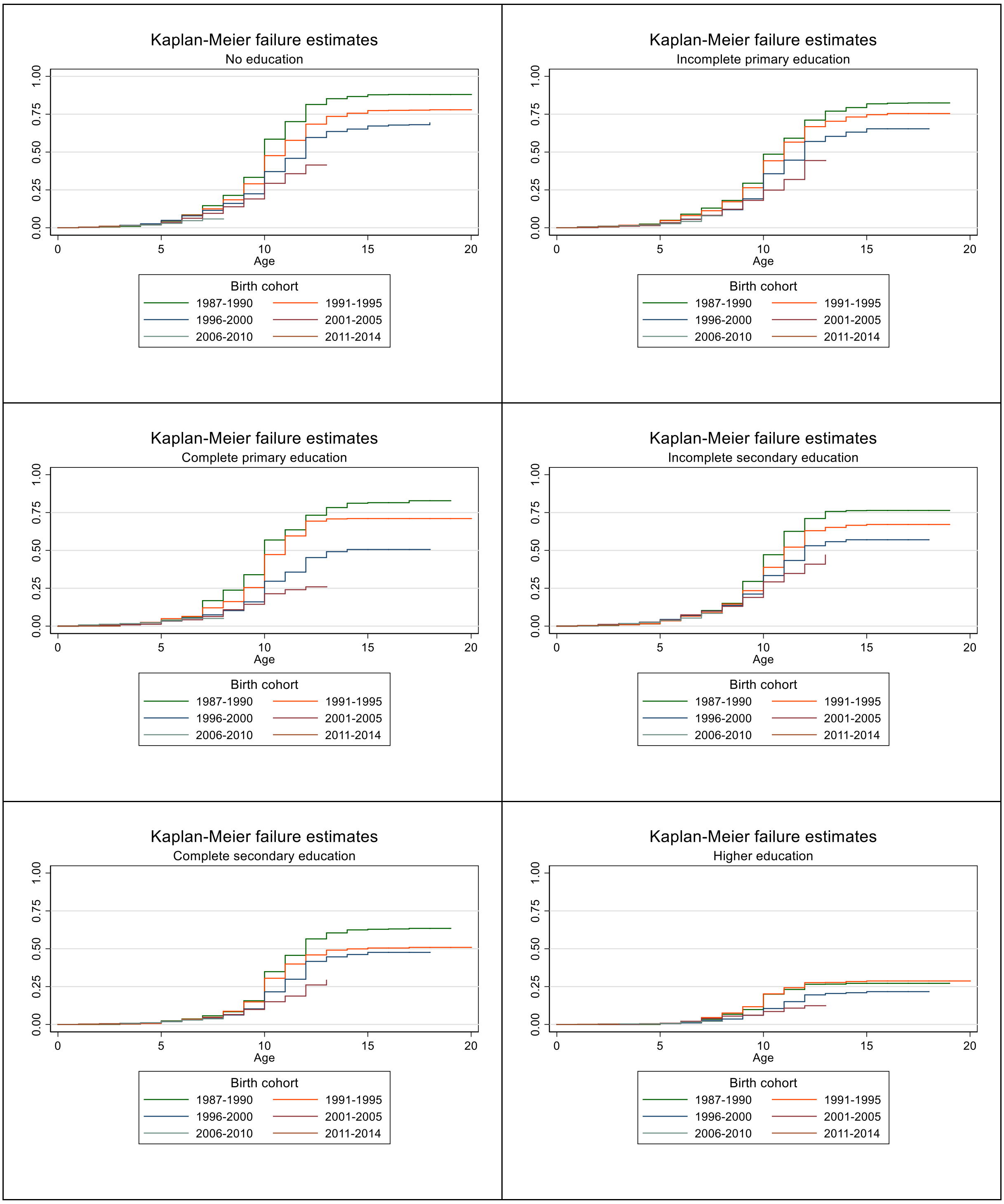

Fig. S2 Kaplan-Meier estimates for FGM by age, birth cohort, and mother's education

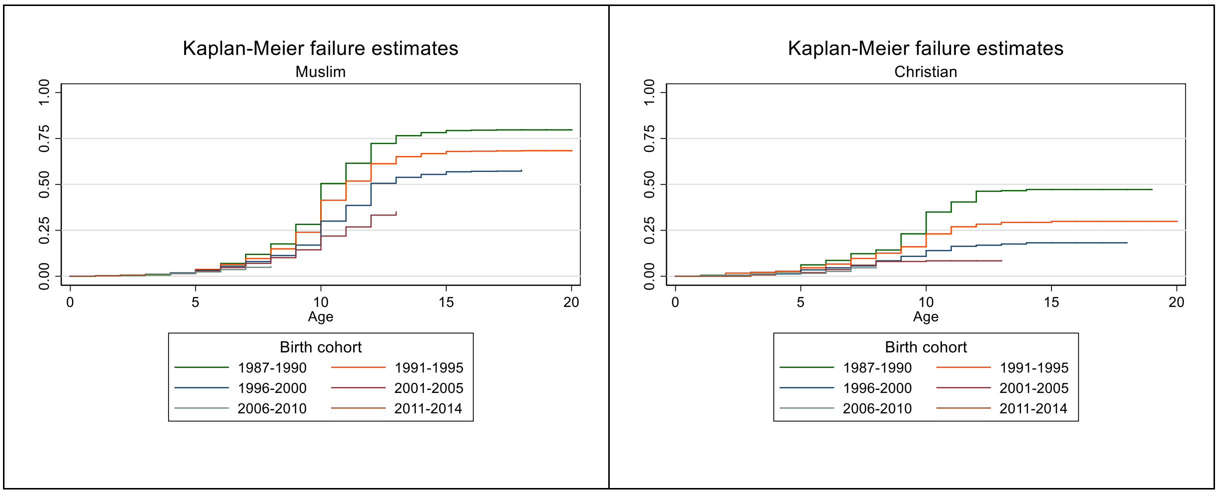

Fig. S3 Kaplan-Meier estimates for FGM by age, birth cohort, and mother's religion

1

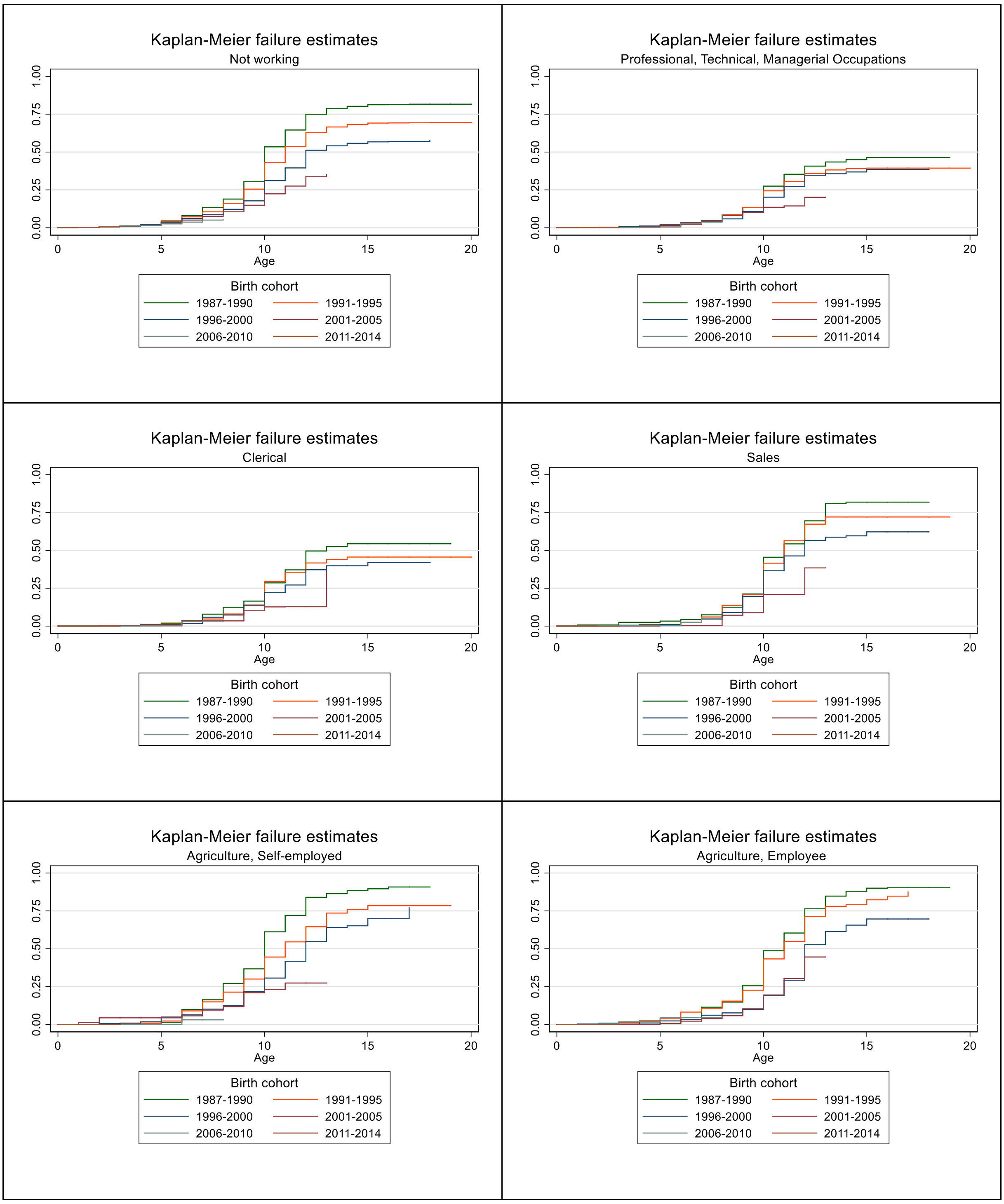

Kaplan-Meier failure estimates
Not working
Kaplan-Meier failure estimates
Professional, Technical, Managerial Occupations
Kaplan-Meier failure estimates
Clerical
Kaplan-Meier failure estimates
Sales
Kaplan-Meier failure estimates
Agriculture, Self-employed
Kaplan-Meier failure estimates
Agriculture, Employee
0.00
0.25
0.50
0.75
1.00
0
5
10
15
20
Age
Birth cohort
1987-1990
1991-1995
1996-2000
2001-2005
2006-2010
2011-2014

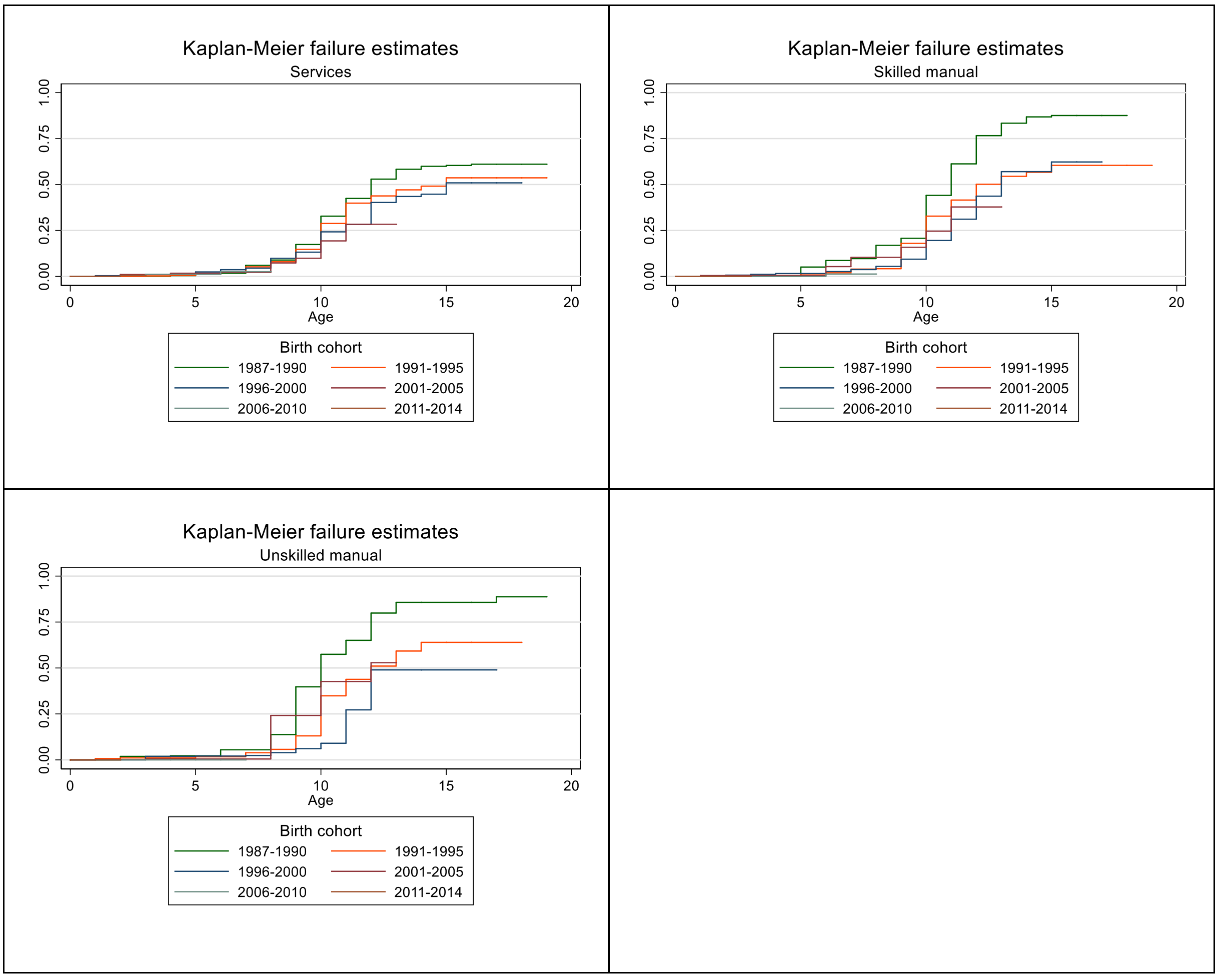

Fig. S4 Kaplan-Meier estimates for FGM by age, birth cohort, and mother's occupation

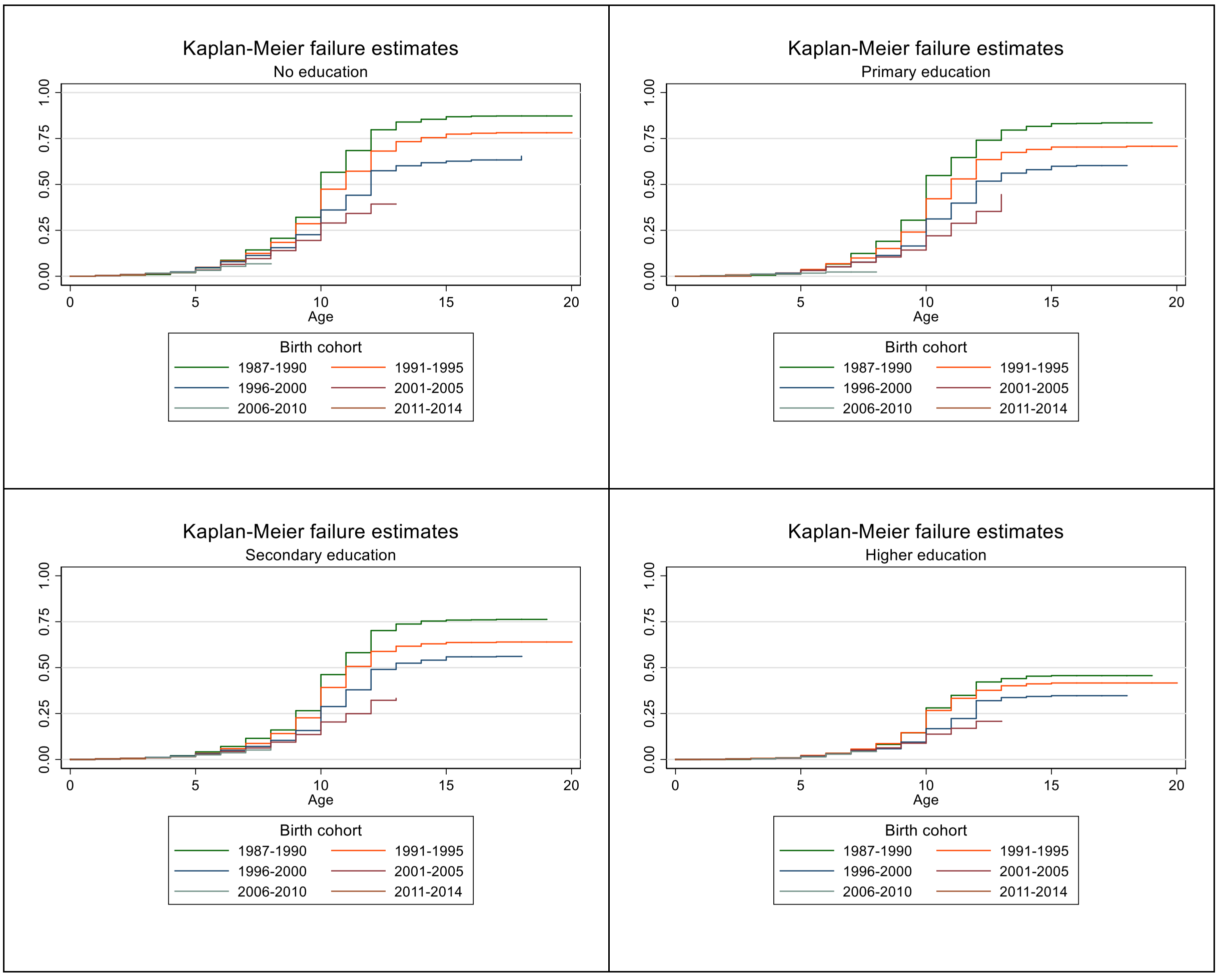

Fig S5 Kaplan-Meier estimates for FGM by age, birth cohort, and father's education

1

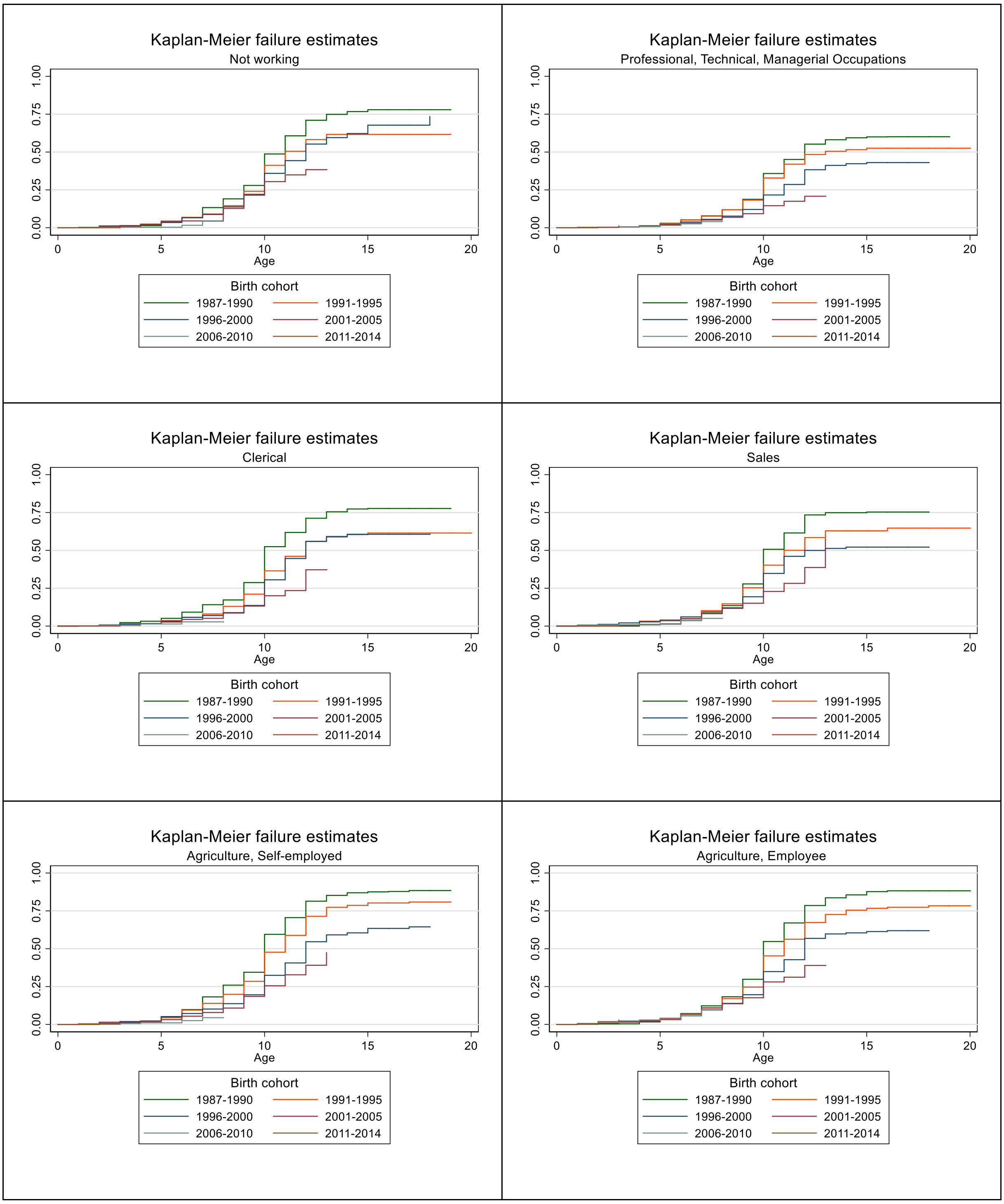
Kaplan-Meier failure estimates
Not working
Kaplan-Meier failure estimates
Professional, Technical, Managerial Occupations
Kaplan-Meier failure estimates
Clerical
Kaplan-Meier failure estimates
Sales
Kaplan-Meier failure estimates
Agriculture, Self-employed
Kaplan-Meier failure estimates
Agriculture, Employee
0.00
0.25
0.50
0.75
1.00
0
5
10
15
20
Age
Birth cohort
1987-1990
1991-1995
1996-2000
2001-2005
2006-2010
2011-2014

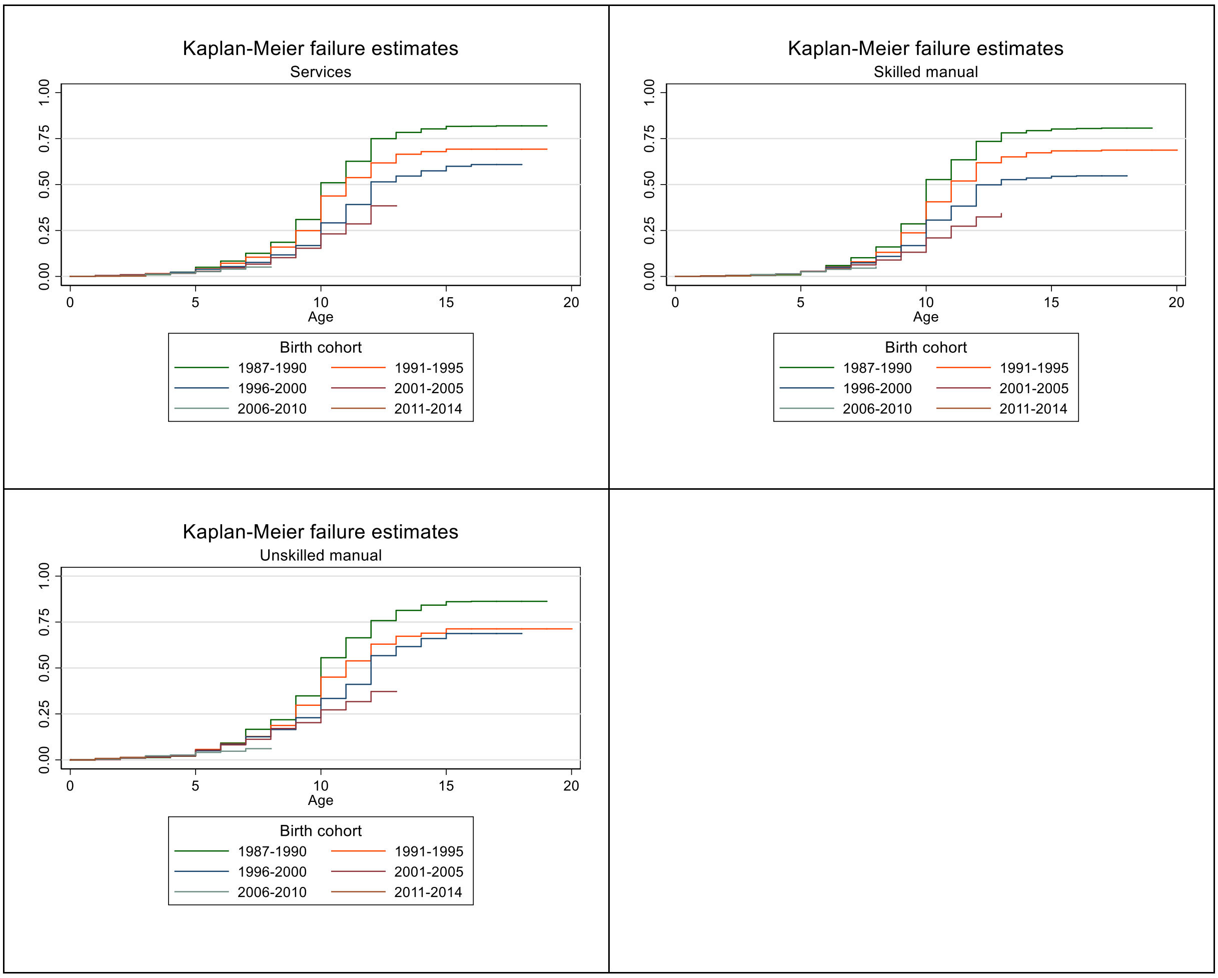

Fig S6 Kaplan-Meier estimates for FGM by age, birth cohort, and father’s occupation
